# CCL11, a novel mediator of inflammatory bone resorption

**DOI:** 10.1038/s41598-017-05654-w

**Published:** 2017-07-13

**Authors:** Elin Kindstedt, Cecilia Koskinen Holm, Rima Sulniute, Irene Martinez-Carrasco, Richard Lundmark, Pernilla Lundberg

**Affiliations:** 1 Department of Odontology/Molecular Periodontology, Umeå University, SE-901 87 Umeå, Sweden; 2 Department of Medical Biochemistry and Biophysics, Laboratory for Molecular Infection Medicine Sweden, Umeå University, SE-901 87 Umeå, Sweden; 3 Department of Integrative Medical Biology, Umeå University, SE-901 87 Umeå, Sweden

## Abstract

Normal bone homeostasis, which is regulated by bone-resorbing osteoclasts and bone-forming osteoblasts is perturbed by inflammation. In chronic inflammatory disease with disturbed bone remodelling, *e.g*. rheumatoid arthritis, patients show increased serum levels of the chemokine eotaxin-1 (CCL11). Herein, we demonstrate an inflammatory driven expression of CCL11 in bone tissue and a novel role of CCL11 in osteoclast migration and resorption. Using an inflammatory bone lesion model and primary cell cultures, we discovered that osteoblasts express CCL11 *in vivo* and *in vitro* and that expression increased during inflammatory conditions. Osteoclasts did not express CCL11, but the high affinity receptor CCR3 was significantly upregulated during osteoclast differentiation and found to colocalise with CCL11. Exogenous CCL11 was internalised in osteoclast and stimulated the migration of pre-osteoclast and concomitant increase in bone resorption. Our data pinpoints that the CCL11/CCR3 pathway could be a new target for treatment of inflammatory bone resorption.

## Introduction

The skeleton is not a static organ as evident by the active process of bone remodelling, which relies on a delicate balance between bone-forming osteoblasts and bone-resorbing osteoclasts^1^. Disruption of the homeostatic balance of bone removal and replacement can manifest as pathologic bone loss, which is observed in *e.g*. osteoporosis, periodontitis, rheumatoid arthritis, peri-implantitis and primary or metastatic bone tumours^2^.

The osteoclast is a cell type of hematopoietic origin, which is formed when osteoclast precursor cells migrate from the blood or the bone marrow into bone tissues where these precursor cells further differentiate and fuse to form a highly motile multinucleated bone-resorbing cell on bone surfaces. Cytokines released by *e.g*. osteoblasts, stromal cells or T-lymphocytes are crucial for osteoclastogenesis. Macrophage colony-stimulating factor (M-CSF) is obligate for survival and proliferation of osteoclast precursor cells^3^. The protein Receptor Activator of Nuclear factor Kappa-B Ligand (RANKL) produced by osteoblasts, T-lymphocytes and endothelial cells is the key cytokine promoting differentiation of mature osteoclasts. Phenotypic markers of mature osteoclasts are tartrate-resistant acid phosphatase (TRAP) and Cathepsin K. Osteoprotegrin (OPG) is a soluble molecule that shares structural similarities with RANK and has the ability to inhibit the interaction between RANK and RANKL and thereby decrease the formation of osteoclasts. Besides these key regulators, additional co-stimulatory molecules including immunoglobulin-like receptors, cytokines, interferons, hormones and chemokines are involved in osteoclast formation by regulating the ratio between RANKL and OPG or by exerting direct effects on osteoclast progenitors^2^.

A locally increased number of osteoclasts at inflammatory sites are dependent on the recruitment of osteoclast progenitors from the blood or bone marrow to the inflammatory tissue. The homing of osteoclast progenitors from the circulation to the surfaces of cortical and trabecular bone is not well understood, but chemokines and lipid mediators are thought to play an important role^4^. Chemokines constitute a superfamily of small, secreted proteins with chemotactic activity, which play a central role in many homeostatic and pathological processes in the body. These molecules were initially described to regulate chemotaxis but subsequent research has pointed to their involvement also in other aspects of the inflammatory process, such as fibrosis, tissue remodelling and angiogenesis. Chemokines exert their biological effects by interacting with G protein-linked transmembrane receptors expressed on the surface of target cells. Thus, an alteration of chemokine and/or chemokine receptor expression might lead to the persistence of an inflammatory reaction well beyond its original purpose, thereby creating a key pathogenic event for the establishment of chronic inflammation, *e.g*. in the gut^5^ or in lung tissue^6^.

Eotaxin-1, encoded by the CCL11 gene and thereby denoted CCL11, is a chemokine within the CC subfamily that is produced by a variety of cell types including endothelial cells, epithelial cells, eosinophils^7^, fibroblasts^8, 9^, keratinocytes^10^, smooth muscle cells^11^ and chondrocytes^12^. CCL11 binds to the chemokine receptors CCR2, CCR3 and CCR5, with highest affinity to CCR3^13, 14^. By interacting with CCR3, CCL11 stimulates the migration of mast cells, eosinophils, Th2- cells, basophils, neutrophils and macrophages^15, 16^. High levels of CCL11 have been described in several chronic inflammatory diseases, such as allergic rhinitis^17^, atopic dermatitis^18^, asthma^6^, gastrointestinal disease^5^ and rheumatoid arthritis^19^. In a recently published study, we could demonstrate higher serum levels of CCL11 in individuals with periodontitis, a chronic inflammatory disease in tooth supporting tissues that if untreated leads to loosened teeth^9^.

Since serum levels of CCL11 are increased in chronic inflammatory disease manifesting with disturbed bone remodelling, and much is unknown regarding the function of CCL11 and its cognate receptor CCR3 in bone tissue, the aim of this study was to analyse CCL11 and CCR3 expression in an *in vivo* inflammatory bone lesion model in addition to also analyse the effect of CCL11 and CCR3 on preosteoclast migration and bone resorption *in vitro*.

## Results

### Pam2 stimulates osteoclast formation, bone resorption, and increased CCL11 expression in mouse parietal bones *in vivo*

**Figure 1:**
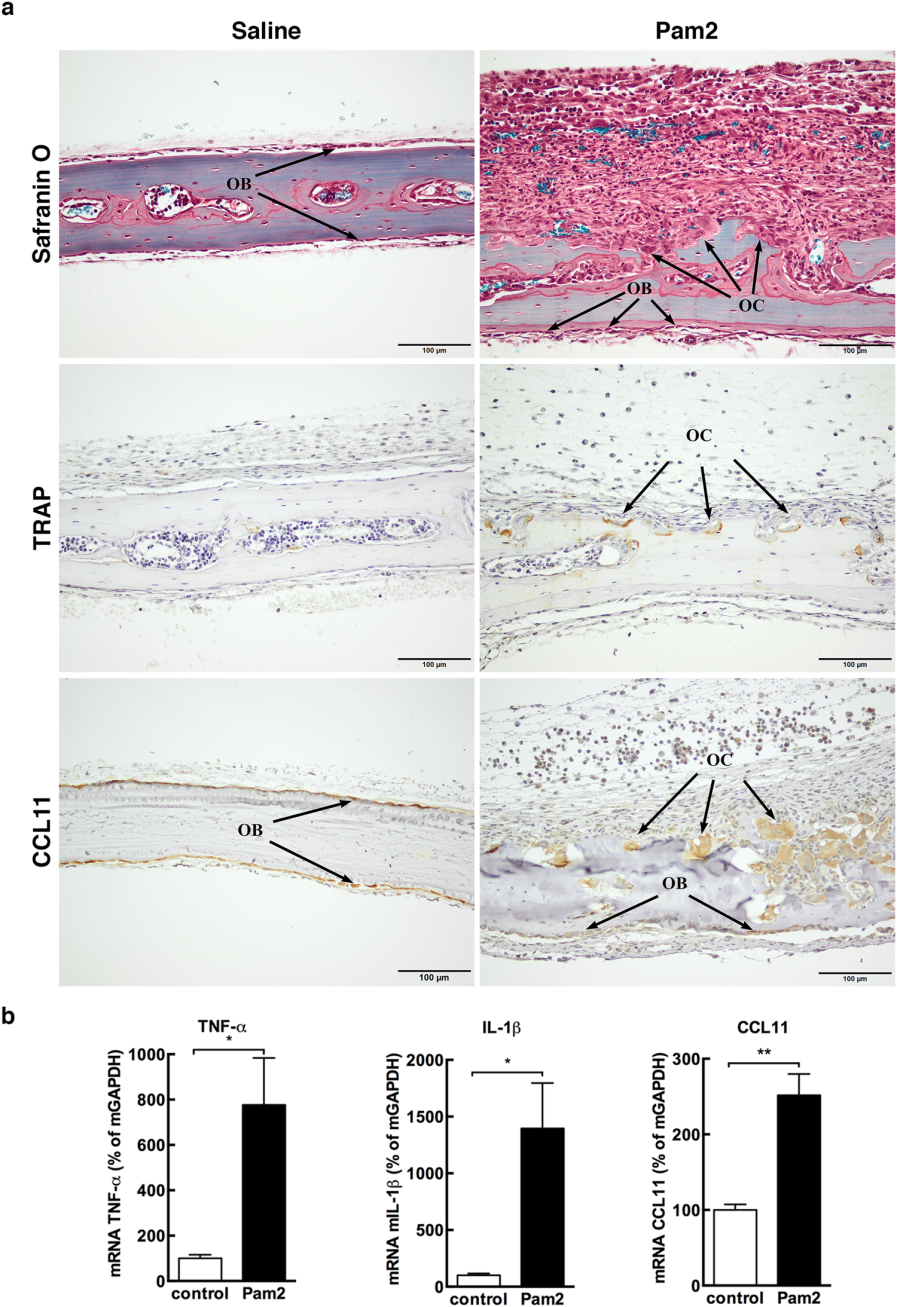
Injection of Pam2 stimulates osteoclast formation, bone resorption and increased CCL11 expression in mouse parietal bones. (**a**) Sections of paraffin embedded parietal bones from mice given subcutaneous injections of saline (left panel) and 50 μg Pam2 (right panel) stained with Safranin O and immunohistochemically for TRAP and CCL11 respectively. Arrows point few osteoblasts (OB) and osteoclasts (OC). (**b**) mRNA expression of TNF-α, IL-1β and CCL11 in homogenized parietal bones from mice subcutaneously injected with Pam2 (n = 4) compared to saline control (n = 5). Data expressed as means ± SEM.

Subcutaneous injection of Pam2CSK4 (Pam2), a synthetic lipopeptide agonist of Toll-like receptor (TLR) 2/6, causes a potent inflammatory response^20^. Therefore, 50 μg of Pam2 were injected subcutaneously above the skull bones of five-week-old CsA mice to induce inflammation. As previously described^20^ this treatment caused extensive bone resorption at day 6 after injection (Fig. 1a), whereas the skull bones from mice injected with saline were unaffected. The presence of osteoclasts in the skull bones of Pam2-injected mice was revealed by immunohistochemical TRAP staining (Fig. 1a). To investigate if Pam2-induced inflammation resulted in local production of CCL11, we next analysed CCL11 protein expression in parietal bone sections using immunohistochemical methods. In the saline-treated group as well as in the Pam2-treated group, CCL11 was detected in all periosteal osteoblasts and in a few cells in the marrow space (Fig. 1a). The multinucleated cells adjacent to bone surfaces in samples from Pam2-treated mice also stained positive for CCL11 (Fig. 1a), and by TRAP staining we could verify that these cells were indeed osteoclasts (Supplementary 1). To further confirm that CCL11 levels were increased by Pam2-induced inflammation, we quantitatively assessed mRNA in homogenates of parietal bones from mice treated with Pam2. We found that the mRNA expression levels of TNF-α, IL-1β and CCL11 were increased approximately 8-fold (*P* < 0.05), 14-fold (*P* < 0.05) and 2.5-fold (*P* < 0.01), respectively, in response to Pam2, as compared with that in saline-injected mice (analysed using Students t-test) (Fig. 1b).

### Increased CCL11 mRNA and protein expression in mouse parietal osteoblasts

**Figure 2:**
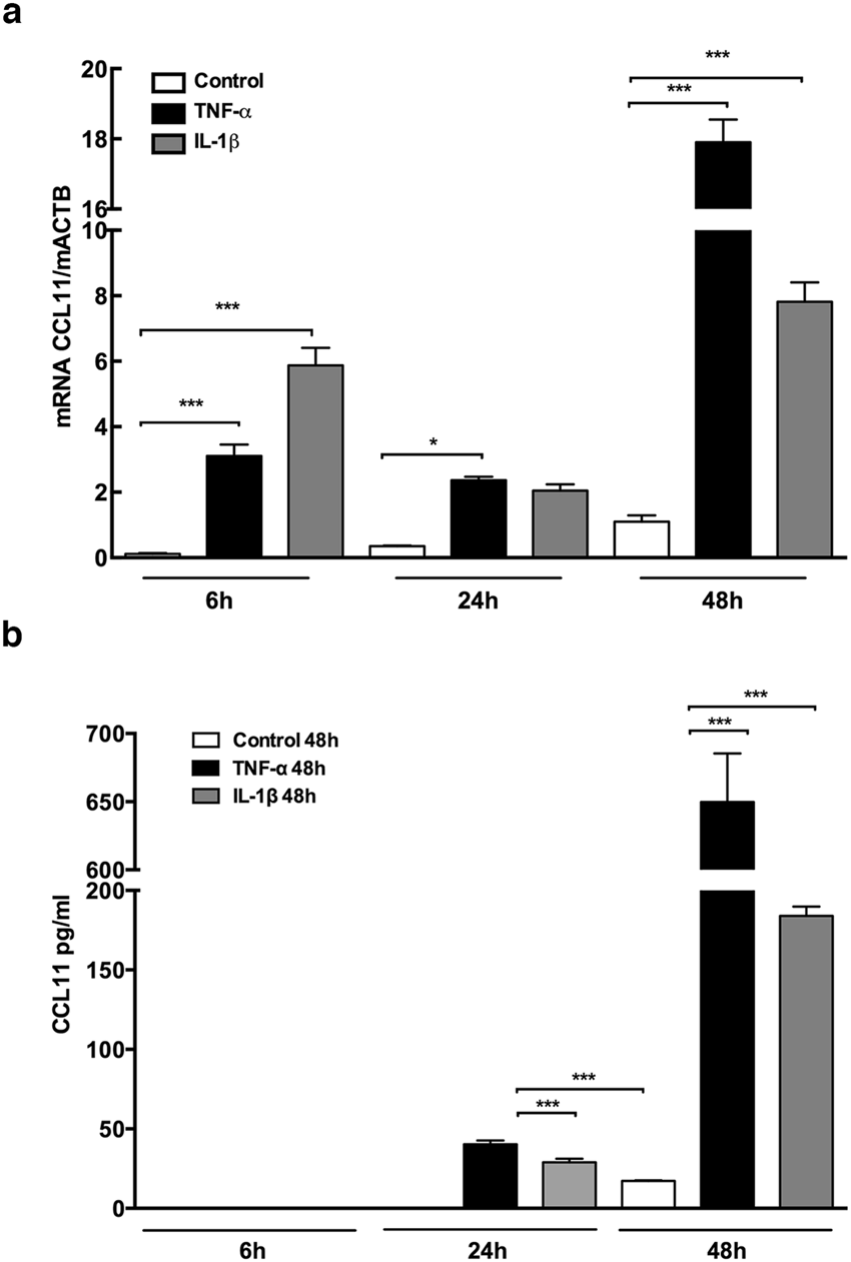
CCL11 mRNA and protein expression in TNF-α and IL-1β stimulated mouse osteoblasts. (**a**) mRNA expression of CCL11 in non-stimulated and TNF-α or IL-1β stimulated osteoblasts after 6 h, 24 h and 48 h of incubation. (**b**) CCL11 release measured in medium cultured in absence and presence of TNF-α and IL-1β after 24 h and 48 h of incubation. Data are expressed as means ± SEM.

To investigated if parietal osteoblasts could contribute to the increased CCL11-levels detected in the bone resorption mouse model, CCL11 mRNA and protein levels were analysed in mouse parietal osteoblasts cultured for 6, 24 or 48 hours in the absence (control) or presence of TNF-α (50 ng/mL) or IL-1β (100 pg/mL). CCL11 mRNA was detected in the control group at 6 h of culture, and both TNF-α and IL-1β significantly increased the expression (28-fold P < 0.001, 53-fold P < 0.001, respectively, analysed using ANOVA) (Fig. 2a). At 24 h of culture, the CCL11 mRNA expression decreased both in response to TNF-α and IL-1β, as compared with that at 6 h, but there was still significantly higher levels of CCL11 mRNA in the TNF-α treated group (7-fold P < 0.05), as compared to control. An increase in the basal CCL11 mRNA expression was seen at 48 h, and both TNF-α and IL-1β were found to upregulate the expression 16-fold (P < 0.001) and 7-fold (P < 0.001), respectively (Fig. 2a). CCL11 protein was undetectable in the culture medium in all groups at 6 h and in the control group at 24 h of culture (Fig. 2b). However, at 24 h CCL11 protein was demonstrated in both the TNF-α and IL-1β treated groups (40.3 pg/mL and 29.0 pg/mL, respectively). At 48 h of culture, CCL11 protein was detected in the control group and TNF-α and IL-1β significantly upregulated the levels (38-fold and 11-fold P < 0.001, respectively, analysed using ANOVA) (Fig. 2b).

### Osteoclasts do not express CCL11 mRNA but their expression of CCR3 mRNA is upregulated by RANKL during osteoclast differentiation

**Figure 3:**
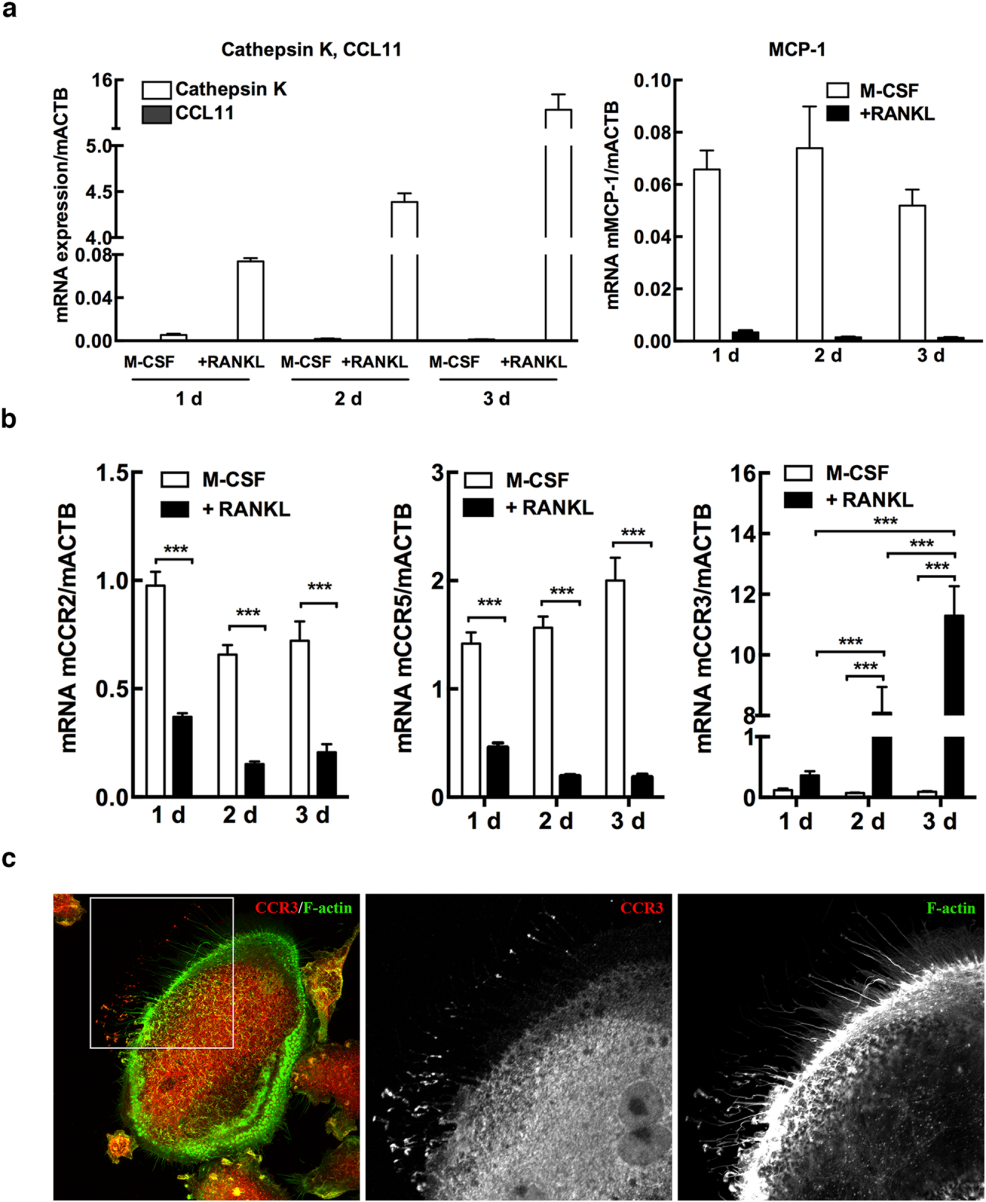
Osteoclasts do not express CCL11 but CCR3 mRNA and protein. (**a**) mRNA of CCL11 and the osteoclast associated enzyme Cathepsin K (left panel) and MCP-1 (right panel) analysed in BMM cultures stimulated with M-CSF alone or in presence of RANKL for 1, 2 and 3 days. (**b**) CCR2, CCR5 and CCR3 mRNA expression during RANKL-stimulated osteoclast differentiation. Data are expressed as means ± SEM. (**c**) Representative immunofluorescence staining of osteoclast culture (day 3) with CCR3 antibody in combination with F-actin to visualize actin filaments.

Since osteoclasts present during inflammatory conditions *in vivo* were found to stain positive for CCL11 (Fig. 1a), the possible expression of CCL11 in osteoclasts during their differentiation was analysed *in vitro* in bone marrow macrophages (BMMs) stimulated with M-CSF (30 ng/mL) and RANKL (4 ng/mL). Quantification of TRAP-positive multinucleated cells confirmed that osteoclast differentiation was increased during the culture time (Supplementary 2), and that the expression of Cathepsin K mRNA was upregulated by RANKL at 2 and 3 days of culture (*P* < 0.001, analysed using ANOVA) (Fig. 3a left graph). CCL11 mRNA expression was undetectable in BMMs cultured for 1–3 days either M-CSF alone or with M-CSF and RANKL (Fig. 3a, left graph). In contrast, mRNA for Monocyte Chemoattractant Protein-1 (MCP-1), a chemokine known to be expressed by macrophages and osteoclasts^21^ was detected in BMMs stimulated with M-CSF but was downregulated in the presence of M-CSF and RANKL (Fig. 3a, right graph). Since CCL11 can bind to the receptors CCR2, CCR3 and CCR5, we next investigated if osteoclast differentiation affected the expression levels of these receptors. Interestingly, the mRNA expression of the high affinity CCL11 receptor CCR3 was very low in the presence of M-CSF alone, whereas it was significantly upregulated by the addition of RANKL at 2 and 3 days of culture (*P* < 0.001, analysed using ANOVA) (Fig. 3b). In contrast, the mRNA levels of CCR2 and CCR5 were significantly downregulated by RANKL, as compared with that in the presence of M-CSF alone (Fig. 3b).

**Figure 4:**
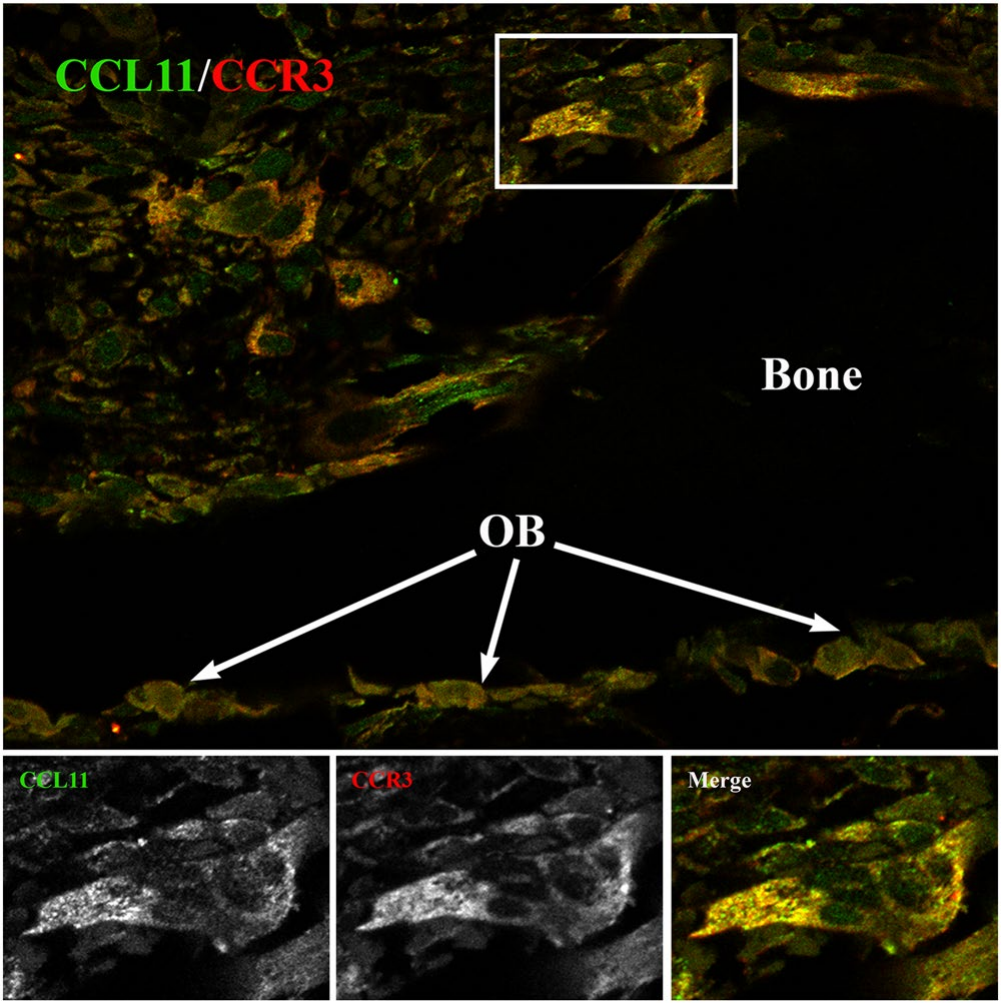
Co-localization of CCL11 and CCR3 receptor in osteoclasts of mouse parietal bones treated with PAM2. Representative immunofluorescence staining of parietal tissue section using CCL11 and CCR3 antibodies. Arrows point the layer of osteoblasts lining up on the bone surface (black). Lower panel demonstrates the higher magnification of squared osteoclast from image above.

To visualise the subcellular localisation of CCR3, differentiated osteoclasts were co-immunolabelled for CCR3 and F-actin. Confocal microscopy revealed that CCR3 was localised to protrusions and ruffled borders in mononuclear osteoclasts (Fig. 3c). Interestingly, in mature multinuclear osteoclasts, CCR3 was detected at the tip of thin actin-rich protrusions polarised to specific areas of the cells, in addition to showing a homogeneous intracellular distribution (Fig. 3c). This polarised localisation of CCR3 suggested that it might be important to sense the environment and guide migration of osteoclasts. To study the possible interaction between CCL11 and CCR3 in tissues, we immunolabelled CCL11 and CCR3 in parietal bone sections from mice treated with Pam2. We found that osteoclasts and osteoblasts stained positive for both CCL11 and CCR3. In osteoclasts, it was apparent that the localisation of CCL11 overlapped with that of CCR3 in discrete areas at the subcellular level. This suggested that CCL11 produced by osteoblasts could interact with CCR3 expressed by osteoclasts (Fig. 4).

### CCL11 binding and effects on osteoclast migration and bone resorption

**Figure 5:**
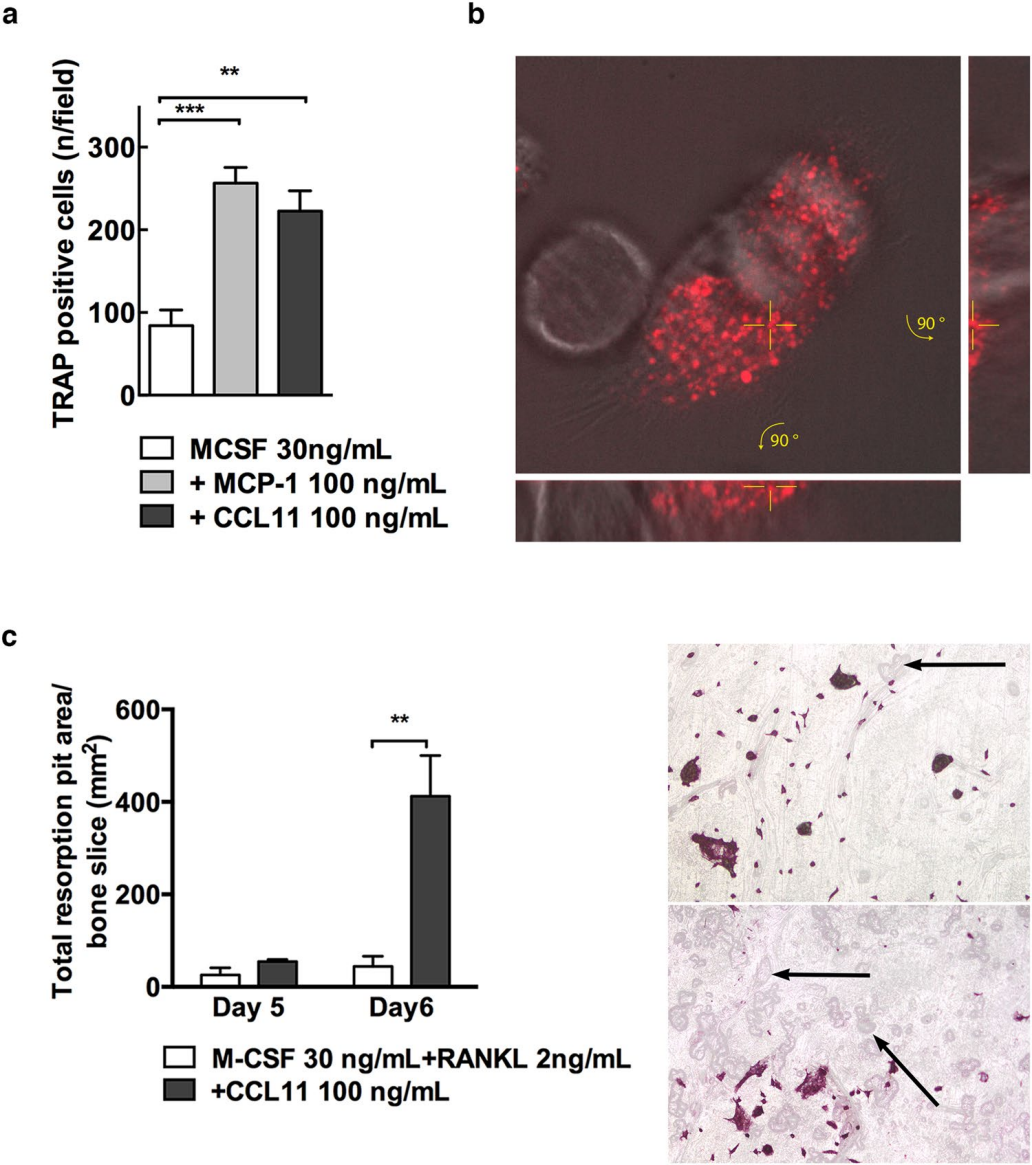
CCL11 binding and effects on osteoclast migration and bone resorption. (**a**) Number of TRAP^+^ cells determined by a chemotaxis migration assay in presence of MCP-1 or CCL11. (**b**) Confocal section of living osteoclasts incubated for 30 min with Alexa Fluor®647 labelled rmCCL11. DIC images were overlaid with the fluorescent images to illustrate the morphology of the cells. Bottom and side projections show slice of the merged maximum projection at position indicated by yellow lines at 90° rotation. (**c**) BMM cultured on bone slices in absence or presence of rmCCL11 in medium supplemented with M-CSF (30 ng/ml) and RANKL (2 ng/ml). Total resorption pit area measured in both groups at day 5 and 6. Data are expressed as means ± SEM. Representative images of resorption pits on bone slices and TRAP^+^ osteoclasts in absence (upper panel) and presence (lower panel) of CCL11. Arrows point resorption pits.

The increased expression of CCR3 receptors during osteoclastogenesis, together with the co-localisation of CCL11 and CCR3 in osteoclasts of inflammatory bone lesions, raised the question whether CCL11 could stimulate chemotactic migration of osteoclast precursors. To investigate this, we used a migration assay where osteoclast precursors were allowed to migrate towards either M-CSF alone or combined with MCP-1 or CCL11. These experiments demonstrated that CCL11 significantly stimulated the migration of osteoclast precursors, as assessed by the number of TRAP^+^ cells trapped in the migration filter (2.2-fold, *P* < 0.01, analysed using students t-test. Fig. 5a). As expected, we found that MCP-1, which is known to stimulate the migration of osteoclast precursors^22^, induced a 2.6-fold increase in osteoclast precursor migration, as compared to that in the presence of M-CSF (*P* < 0.001, analysed using students t-test Fig. 5a).

To visualise the interplay between CCL11 and osteoclasts, we added fluorescently labelled CCL11 to cultured osteoclasts and recorded live cell movies using DIC and spinning disc microscopy. Following 5-15 minutes of incubation, CCL11 was starting to cluster in distinct punctae in the cells (Supplementary 3A). After 40 minutes of incubation, we observed a striking accumulation of CCL11 in internal structures within both mono- and multinucleated osteoclasts (Fig. 5b and Supplementary 3B). These findings showed that CCL11 was efficiently internalised in cultured osteoclasts.

Next, we analysed if the bone resorption pattern of osteoclasts was altered in the presence of CCL11. The total area of resorption pits on bone slices, formed by osteoclasts stimulated with M-CSF (30 ng/mL) and RANKL (2 ng/mL) with and without the addition of CCL11 (100 ng/mL) was measured at 5 or 6 days of culture. At 5 days of culture, CCL11 gave a non-significant 2-fold increase in the total pit area, as compared to BMM-cultures with only M-CSF and RANKL. In contrast, the total pit area was significantly increased at 6 days of culture (9-fold, *P* > 0.01, analysed using students t-test) in bone slices incubated with CCL11, as compared to control cultures (Fig. 5c). No difference in the number of multinucleated osteoclast in the culture groups was detected (Supplementary 4).

## Discussion

Osteoclastic bone resorption is often a sequel of osseous inflammation and jeopardise loss of function in the afflicted tissues. Although various drugs have been developed, there are currently no established methods for effective prevention of bone destruction in diseases like periodontitis and RA or in bone invading tumour processes. In this study, we discovered that osteoblasts *in situ* and *in vitro* express CCL11 and found that the expression increased during inflammatory conditions. Further analysis showed that CCL11 was colocalised with its high affinity receptor CCR3 in osteoclasts under conditions of inflammatory bone resorption *in vivo*. Supporting our *in vivo* findings, we could demonstrate that RANKL stimulated CCR3 expression in osteoclasts *in vitro* and that addition of CCL11 caused an increased migration of osteoclast precursors and an increase in osteoclastic bone resorption.

Chemokines and chemokine receptors have been shown to regulate bone metabolism^2^. CCL3 and its cognate receptor CCR1 is one of the best documented. CCR1-deficiency affects the differentiation and function of both osteoblasts and osteoclasts, and also causes osteopenia^23^. Most interestingly, osteoblasts from CCR1 deficient mice expressed lower levels of CCL11 then normal osteoblasts, and had a reduced *in vitro* mineralisation capacity (Hoshino). This implies a possible chemokine-dependent amplification loop in bone metabolism.

The recruitment of osteoclast precursors towards bone-lining osteoblasts expressing RANKL on their cell surface is critical for osteoclast differentiation. Recent studies have revealed the involvement of several chemokines in controlling osteoclast precursor migration from the blood into bone tissues, or in controlling their migration within the bone cavity. One of the best characterised chemoattractants controlling osteoclast precursor migration is stromal cell-derived factor-1 (CXCL12/SDF-1)^24, 25^. CXCL12 is highly expressed by osteoblasts as well as by specific stromal cells enriched in perivascular regions in the bone marrow cavity. On the other hand, the CXCL12 receptor CXCR4 is expressed on a wide variety of haematopoietic cells, including circulating monocytes and osteoclast precursors. CXCL12 has been shown to promote chemotactic recruitment, development and survival of osteoclast precursors^26^.

CCL11 has been found to play a crucial role in recruitment of leukocytes such as mast cells, eosinophils, Th2- cells, basophils, neutrophils and macrophages by binding to the receptor CCR3. CCR3 has a very high affinity for CCL11, but is also able to bind other chemokines, including RANTES, MCP-2, MCP-3 and MCP-4. Our finding of both a constitutive and an inflammatory stimulated osteoblastic expression of CCL11 pinpoint this chemokine as a novel player in physiological as well as pathological bone remodelling. In our *in vivo* inflammatory mouse model, we could show that mononucleated osteoclast precursors near bone surfaces, and multinucleated osteoclasts sitting on bone surfaces and covering resorption lacuna, expressed CCR3 receptors which co-localized with CCL11. This, together with our finding that CCL11 increased pre-osteoclast migration *in vitro* indicate that the CCL11/CCR3 axis could be of importance for migration of pre-osteoclasts to bone surfaces. Moreover, we show that RANKL stimulates CCR3, and down-regulates CCR2 and CCR5 mRNA expression in osteoclast cultures. This is in line with earlier reports^27, 28^ and indicates that inflammation stimulate the CCR3 expression. Interestingly, an upregulated expression of CCR3 receptor has been found in osteoarthritis cartilage and on human chondrocytes indicating that CCR3 play a role in inflammatory cartilage destruction^29^.

Binding of CCL11 and subsequent activation of CCR3 is thought to occur via a two-step model in which a high affinity interaction between the core residues of the chemokine and the N-terminus of the receptor initially tethers CCL11 to CCR3. This facilitates subsequent interaction between the chemokine and the remainder of receptor leading to signalling^30^. In this report we show that CCL11 was bound and efficiently internalised in cultured mono- and multinucleated osteoclasts. CCR3 is a 7-transmembrane G protein-coupled receptor and induce a variety of downstream signals, which notably modulate polymerization of the actin cytoskeleton and thus drive cellular motility^31^. Therefore, it is very intriguing that we found CCR3 at the tip of thin actin-rich protrusions on the polarised osteoclast cell membrane suggesting that CCR3 could affect migration of osteoclasts.

Osteoclasts are highly motile cells, in which specific organisation of their most prominent cytoskeletal structures, podosomes, is crucial not only for the migration but also for the degradation of mineralized bone matrix. Each podosome is constituted of an F-actin-enriched central core surrounded by a loose F-actin network. To resorb bone, osteoclasts polarise, actively secrete protons and proteases into the sealed area and a resorption pit is created^32^. Our finding that addition of CCL11 to osteoclasts cultured on bone slices increased the total resorption pit area could be due to that fact that CCL11 binds to F-actin associated receptors, which signalling affect podosome activity. Further studies are needed to elucidate if the increased resorption area caused by CCL11 is a result of increased osteoclast motility, resorptive activity or both.

In summary, we have found that the chemokine CCL11, together with its cognate receptor CCR3, are associated with disturbed bone remodelling. Furthermore, we found that CCL11 stimulates migration of osteoclast precursors and stimulates bone resorption. Therefore, these findings may have important implications for the development of new anti-osteoclastic therapeutics in inflammatory bone related disorders. Bertilimumab, a humanized monoclonal antibody against CCL11, is currently in clinical trials for treating severe allergic disorders, keratoconjunctivitis, and inflammatory bowel disease^33, 34^. Our present study highlights that the development of therapeutics that influence the regulation of the CCL11/CCR3-axis could be of major importance for the treatment of osseous inflammation.

## Methods

### Materials

Recombinant mouse M-CSF, RANKL and CCL11 were purchased from R&D Systems; the kit for TRAP staining Sigma Aldrich; α-modification of Minimum Essential Medium (α-MEM), fetal bovine serum (FBS), L-glutamine and antibiotics (streptomycin, penicillin and gentamycin) from Life Technologies Ltd., CORNING® 430591 dishes from Corning Inc.; ChemoTx® plate with 8 μm pore size (Neuroprobe); bone slices (IDS immunodiagnostic systems, UK); mouse CCL11 ELISA kit (Abcam); oligonucleotide primers and probes, High-Capacity cDNA Reverse Transcription Kit and TaqMan® Universal PCR Master Mix from Applied Biosystems, Thermo Fisher Scientific; RNAqueous-Micro Total RNA Isolation Kit from Life Technologies; Pam2CSK4 (Pam2) from Invivogen; primary antibodies for CCL11 and CCR3 provided by R&D systems; antibodies against TRAP from Santa Cruz Biotechnology; protein block (serum-free) and polyclonal HRP conjugated secondary antibodies provided by Dako; RNA isolation reagent TRIzol®, Alexa Fluor®488 phalloidin (F-actin), secondary antibodies Alexa Fluor®488 and Fluor®594 from Thermo Fisher Scientific; 3,3′-diaminobenzidine (DAB) from Vector Laboratories.

### Mice

CsA mice from our own inbred colony were used for all experiments involving mice. Animal care and experiments were approved and conducted in accordance with regulations of the Local Animal-Ethical Committee at Umeå University.

### Bone Marrow Macrophage Culture (BMM)

Bone marrow cells were isolated from femurs and tibiae of 5–8 weeks old male CsA mice as described previously^35^. Briefly, after lysis of erythrocytes, cells were placed on 60 cm^2^ culturing dishes (Corning) in α-MEM medium supplemented with 10% FBS, L-glutamine and antibiotics (this will be referred to as a complete medium) and M-CSF (30 ng/ml) each. After 48 h of incubation, non-adherent cells were discarded and remaining adherent macrophages were collected. From this step forward, further procedures depended on the purpose of the BMMs (see osteoclast formation, resorption on bone slices and migration).

### Osteoclast formation and gene expression

BMMs from above mentioned procedure were pelleted and suspended in complete medium. The cells were then seeded at a density of 1 × 10^4^ cells/cm^2^ in culturing plates (Nunc) with complete medium supplemented with M-CSF (30 ng/ml) ± RANKL (2, 3 or 4 ng/ml). The cells were cultured for different periods of time, as indicated by the figure legends. After culture the cells were either stained for TRAP (osteoclast formation) or committed to RNA isolation and cDNA synthesis (gene expression).

### Osteoclast formation on bone slices and bone resorption assessment

BMMs were seeded on bone slices in 96-well plates (Nunc) in complete medium supplemented with M-CSF (30 ng/ml), RANKL (2 ng/ml) ± CCL11 (100 ng/ml). After 5 and 6 days, all bone slices were TRAP-stained the number of TRAP positive cells with three or more nuclei were counted as osteoclasts and the size and area of resorption pits was measured in reflected light^36^.

### TRAP staining

TRAP staining of cells on plastic, bone slices and migration plates was performed using the leukocyte acid phosphatase kit according to protocol provided by the manufacturer. TRAP^+^ cells with three or more nuclei were considered as osteoclasts. The number of multinucleated osteoclasts was counted in a light microscope (Olympus, BX41).

### Migration assay

BMMs were pelleted and suspended in complete medium supplemented with M-CSF (30 ng/ml) and RANKL (4 ng/ml). The cell suspension was added to a new culture dish 60 cm^2^ (Nunc) and the cells were allowed to adhere before adding the remaining medium. After 2 days of incubation cells were washed once with PBS and detached with a cell-scraper in complete medium. The cell suspension was pelleted and re-suspended in complete medium. Medium supplemented with M-CSF (30 ng/ml) and MCP-1 (100 ng/ml) or CCL11 (100 ng/ml) in the test groups was placed to the bottom of each well on a 96-well ChemoTx plate with 8 μm pore size before 7 500 cells in 50 μl medium were added to each filter. After 5 h of incubation the excess of cells was removed from the top of the filter using a cell-scraper. The filters were detached from the plate, fixed and stained for TRAP. The number of TRAP^+^ cells was determined using a light microscope (Olympus, BX41).

### Isolation and culture of primary mouse parietal osteoblast cells

Skull bones from 2–4 days old mice were dissected aseptically in PBS containing 10% FBS and cells were isolated using the time sequentional collagenase digestion technique as previously described^37^. Briefly, cells pooled from 6^th^ to 10^th^ digestions fractions were placed in a 175 cm^2^ culture bottle and incubated for 4 days. At the fourth day the cells were detached with trypsin and transferred onto 24-well plates at a density of 5 × 10^4^ cells/cm^2^. The cells were allowed to adhere overnight, medium was changed and cells were incubated in the absence (control) or presence of the test substances TNF-α (50 ng/ml) or IL-1β (100 pg/ml) for different time periods, as indicated by the figure legends. CCL11 protein concentration was assessed in cell culture supernatants using ELISA kit and cell lysates were subjected to RNA-isolation.

### RNA-isolation and first-stranded cDNA-synthesis

Total RNA from cell cultures was isolated and DNAse treated using the RNAqueous™ Micro Kit, according to instructions provided by the manufacturer. High Capacity cDNA Reverse Transcription Kit was used to transcribe mRNA to cDNA.

### Quantitative Real-Time PCR (RT-qPCR)

Taq-man (ABI PRISM 7900HT Sequence Detection System) was used to detect and analyse gene expression. The mRNA levels of Cathepsin K, TNF-α, IL-1β, CCL11, CCL2 (encoding MCP-1), CCR2, CCR3 and CCR5 were determined using specific primers/fluorescent probe mix. To control variability in amplification, mouse β-actin or GAPDH was used as a housekeeping gene. All samples were run in duplicates. The relative expression of target mRNA was computed from the target Ct values and β-actin or GAPDH Ct values using the standard curve method (User Bullentin #2, Applied Biosystems).

### Pam2-induced inflammation and bone resorption in mouse parietal bone

5-week-old male CsA mice were injected with 100 μl of Pam2 (50 μg) or saline (control group) subcutaneously above the skull bone as described previously^38^ and sacrificed after 6 days. The parietal bones were carefully dissected and either committed to homogenization for RNA isolation or fixation for histology and immunohistochemistry.

### Homogenization and RNA isolation of skull bones

After dissection, parietal bones were homogenized in RNAse free tubes with beads (Navye1-RNA) with TRIzol using the Bullet Blender Storm homogenizer (Next Advance). After chloroform step total RNA isolation was performed according to protocol provided by manufacturer (RNAeasy kit). After DNAse treatment, RNA was synthesized into cDNA as previously described.

### Histology

Dissected parietal bones were fixed in 4% phosphate-buffered paraformaldehyde (PFA), decalcified in 10% EDTA (pH 6.95) and embedded in paraffin. Tissue sections (5 μm-thick) were deparaffinised in xylene, hydrated through a series of graded ethanol-water dilutions and stained with Safranin O for histology.

### Immunohistochemical staining and imaging

Tissue sections were prepared as described above. After sodium citrate buffer heat-induced epitope retrieval step parietal sections were blocked with serum-free blocking solution. BMMs cultures were fixed in 2% PFA, permeabilised with 0,05% Triton X-100 and incubated with blocking solution. After blocking step both tissue sections and BMMs were incubated with primary antibodies: goat polyclonal TRAP, goat polyclonal CCL11 and/or rat monoclonal CCR3 primary antibodies. After wash steps sections and cells were incubated respectively with F-actin, fluorescent secondary antibodies Alexa Fluor®488 or 594 in addition to DAPI or HRP-labelled secondary antibodies visualized with 3,3′-diaminobenzidine (DAB) and followed by a nuclear counterstaining with haematoxylin. Confocal images were acquired using an A1 R Laser Scanning Confocal Microscope system (Nikon Instruments) under control of the NIS-Elements Microscope Imaging Software and a 60X lens (Apochromat 1.40 Oil λS 0.17 WD 0.14, Nikon), at the appropriate excitation and emission wavelengths. Recombinant mouse CCL11 was labelled with Alexa Fluor®647 using NAP5 gravity columns (GE Healthcare Life Sciences) and used for live cell imaging of osteoclast culture. Before imaging osteoclasts grown on glass coverslips were transferred to the Attofluor® cell chamber (Invitrogen), culture media was replaced with DMEM high glucose with HEPES without phenol red supplemented with pyruvate (Gibco) before addition of labelled CCL11. Live cell imaging was performed under controlled conditions of CO_2_ (5%) and temperature (37 °C) with a 100X lens (alpha Plan-Apochromat 100X/1.46) Zeiss Cell Observer Spinning Disk Confocal controlled by ZEN interface with an Axio Observer.Z1 inverted microscope, equipped with a CSU-X1 A 5000 Spinning Disk Unit and a EMCCD camera iXon Ultra from ANDOR.

### Statistics

The statistical analyses were performed using students t-test or were appropriate one-way analysis of variance (ANOVA) with Tukeys test for multiple comparisons. All experiments were performed at least twice. Data are presented as means ± standard error of means (SEM). The significance levels were set to P < 0.05 (*), P < 0.01 (**) and P < 0.001 (***).

### Data availability

The data that support the findings of this study are available from the corresponding author upon request.

## Electronic supplementary material

Supplementary information
