# Supplementary material for: CCL11, a novel mediator of inflammatory bone resorption
Source: Sci Rep. 2017 Jul 13;7:5334. doi: 10.1038/s41598-017-05654-w (PMC5509729; doi:10.1038/s41598-017-05654-w)

CCL11, a novel mediator of inflammatory bone resorption

Elin Kindstedt[1], Cecilia Koskinen Holm[1], Rima Sulniute[1], Irene Martinez-Carrasco[2], Richard Lundmark[2, 3], Pernilla Lundberg[1*]

1. Department of Odontology/Molecular Periodontology, Umeå University, SE-901 87 Umeå, Sweden
2. Department of Medical Biochemistry and Biophysics, Laboratory for Molecular Infection Medicine Sweden, Umeå University, SE-901 87 Umeå, Sweden
3. Department of Integrative Medical Biology, Umeå University, SE-901 87 Umeå, Sweden

***Correspondence:** Pernilla Lundberg, DDS, PhD
Department of Molecular Periodontology
Umeå University, SE- 901 87 UMEÅ, Sweden
Phone: +46 90 785 6294
Mobile phone: +46 70 549 5356
E-mail: pernilla.lundberg@umu.se

Key words: CCL11, CCR3, inflammation, osteoblasts, osteoclasts, bone resorption

SUPPLEMENTARY INFORMATION

S1

Representative immunohistological staining of parietal bone consequent sections using Cathepsin K antibody.

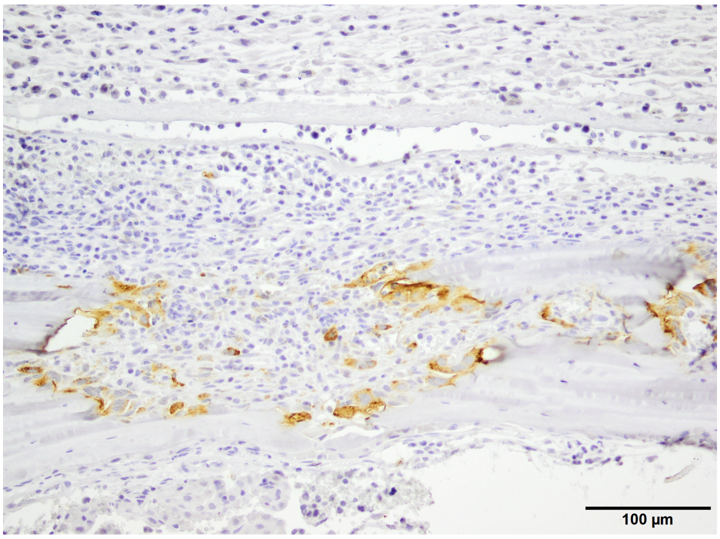

S2

Quantification of TRAP^{+} multinucleated (osteoclasts) cells on culturing plastic, at different time points during culture. Data is expressed as means ±SEM

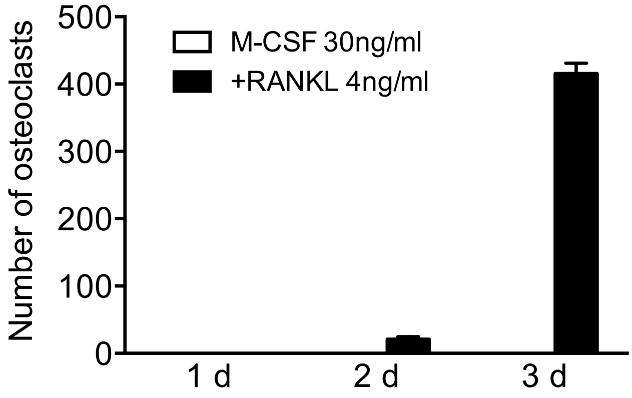

S3

(A) DIC and fluorescent micrographs from live cell movie of osteoclasts incubated with Alexa Fluor®647 labelled rmCCL11 for the time points indicated. Top panel show overlay between DIC images and fluorescent images. (B) Representative images from live cell imaging of osteoclasts incubated for 40 min with Alexa Fluor®647 labelled CCL11. The DIC image was overlaid with the fluorescent image to illustrate the morphology of the cells.

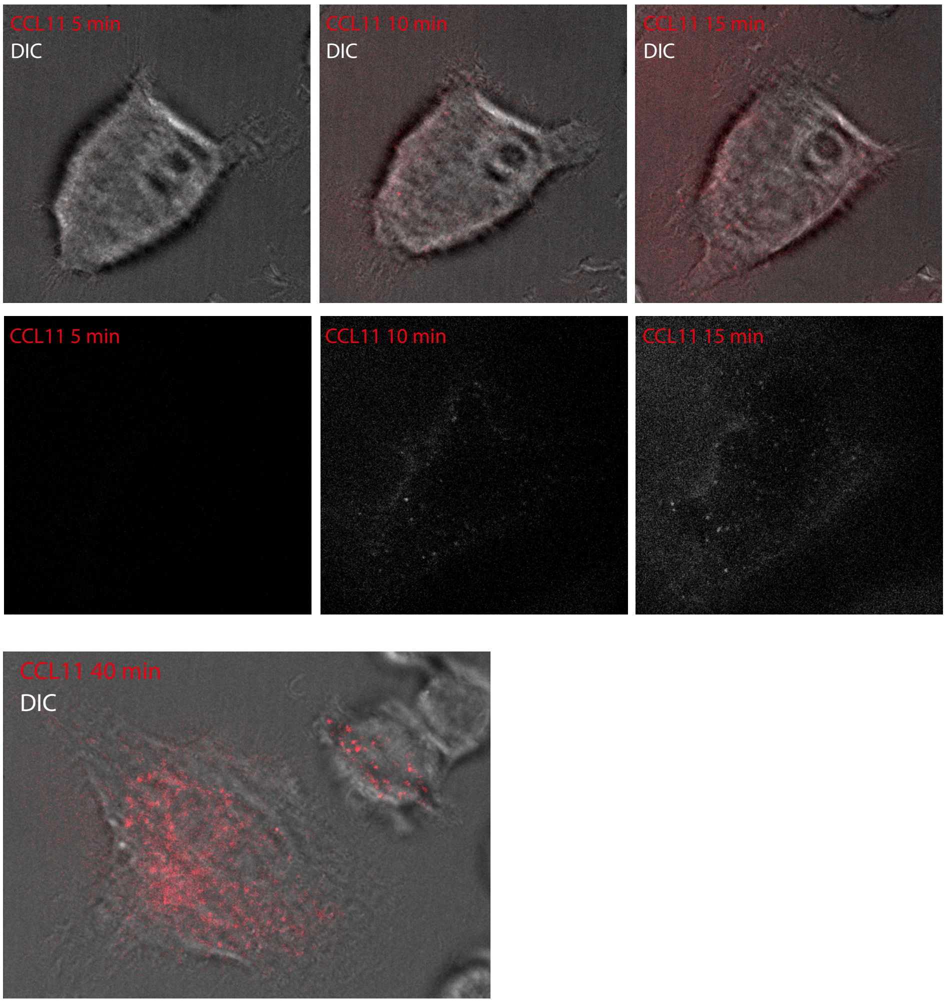

S4

Quantification of number of TRAP+ cells (osteoclasts) after 5 and 6 days of culture, with or without addition of CCL11. Data is expressed as means ±SEM.

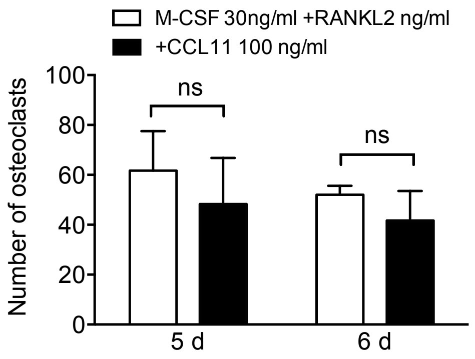

Supplement: Supplementary file 1 — Supplementary information [file 41598_2017_5654_MOESM1_ESM.pdf]
